# Mantle Cell Lymphoma Presenting as Cholecystitis and Nephromegaly

**DOI:** 10.7759/cureus.50536

**Published:** 2023-12-14

**Authors:** Kamen W Kossow, Joseph G Bennett, Wei Cui, Aung M Tun, Robert J Kribs

**Affiliations:** 1 Internal Medicine, University of Kansas Health System, Kansas City, USA; 2 Hematologic Malignancies and Cellular Therapeutics, University of Kansas Health System, Westwood, USA; 3 Pathology and Laboratory Medicine, University of Kansas Health System, Kansas City, USA

**Keywords:** nephromegaly, renal, cholecystitis, gallbladder, mantle cell lymphoma

## Abstract

Mantle cell lymphoma (MCL) most commonly presents as lymphadenopathy (LAD), fevers, night sweats, weight loss, splenomegaly, and blood count abnormalities. While extranodal involvement as an initial presentation can occur, it is uncommon. At initial diagnosis, MCL most commonly presents as widespread, advanced stage III or IV lymphoma. Given advanced stage MCL at presentation, it is important for medical practitioners to recognize unusual extranodal presentations of MCL for earlier diagnosis and treatment planning. Here, we present a case of MCL initially presenting as cholecystitis and bilateral nephromegaly in a 53-year-old male patient.

## Introduction

Similar to all subtypes of lymphoma, mantle cell lymphoma (MCL) most commonly presents with constitutional “B-symptoms” (fevers, night sweats, weight loss), lymphadenopathy (LAD), and blood count abnormalities [1]. While it is less common, extranodal involvement of MCL can be the initial presentation and most commonly involves the spleen, bone marrow, and gastrointestinal (GI) tract [1]. When presenting with extranodal involvement, it is determined to be stage IV, consistent with the frequently advanced stage of MCL at initial diagnosis [2]. 

The frequency of GI involvement of MCL has previously been reported to be 15-30%, with the lower GI tract being the most common site of GI involvement (53.6% lower GI tract involvement vs. 14.3% upper GI tract involvement) [3,4]. Most commonly, GI involvement is characterized by nodules or polyps present on endoscopic evaluation. Other studies have demonstrated even higher rates, reporting the presence of GI tract involvement in up to 90% of patients with MCL who underwent routine endoscopic evaluation [3-6]. While the stomach and intestines are the most common sites of GI involvement in MCL, involvement of the gallbladder is exceedingly rare in any type of lymphoma, including MCL [7]. In a retrospective review of lymphoma cases at the National Institute of Health, the largest case series of gallbladder lymphomas to date found only 19 total cases of gallbladder involvement [7]. Of the 19 cases identified, only one was found to be MCL, with extranodal marginal zone lymphoma, diffuse large B-cell lymphoma, follicular lymphoma, and lymphoblastic lymphoma being more commonly associated with gallbladder involvement [7]. Of these 19 cases, 92% presented with symptoms of cholecystitis, cholelithiasis, and/or jaundice [7]. A literature review was performed and found 36 reported cases of lymphoma involving the gallbladder, though none of these 36 cases were found to be MCL [7].

Similar to gallbladder involvement of lymphoma, direct kidney involvement of lymphoma is extremely rare. A retrospective review over an 18-year time period at two tertiary hospitals identified 20 total cases of patients with renal parenchymal infiltration of lymphoma [8]. Of the 20 cases identified, only one was found to be MCL, with diffuse large B-cell lymphoma, follicular lymphoma, and marginal zone lymphoma being more commonly associated with parenchymal involvement of the kidneys [8]. Of the 20 cases, 14 patients (70%) presented with acute kidney injury and one patient (5%) presented with bilateral nephromegaly [8]. In patients presenting with lymphomatous involvement of the kidneys, an elevated serum creatinine has been reported in up to 56% of patients [9]. Literature review has shown that with lymphomatous involvement of the kidneys, the most common finding on imaging is the presence of multiple parenchymal nodular masses, occurring in approximately 50-60% of cases [9]. Less commonly noted on imaging is the presence of nephromegaly, which almost always occurs bilaterally, and is present in only 6-19% of cases [9].

Overall, gallbladder involvement of MCL is exceptionally rare and has only been reported in case studies [10-12]. Similarly, renal involvement of MCL is also exceptionally uncommon and typically only reported in case studies [13-15]. Here, we present a rare case of a 53-year-old man presenting with both cholecystitis and bilateral nephromegaly secondary to newly diagnosed MCL.

## Case presentation

A 53-year-old male with a past medical history of type 2 diabetes mellitus, hypertension, and hyperlipidemia presented to the emergency department with a three-week history of progressive right upper quadrant abdominal pain with associated nausea and vomiting. Of note, he did not have constitutional symptoms at the presentation. Upon arrival, he was found to have acute kidney injury with a creatinine of 3.73 milligrams per deciliter (mg/dl) (reference range: 0.4 - 1.24). He had no known history of renal disease and had a normal creatinine at baseline. He was oliguric upon initial evaluation. Additionally, the patient’s hemoglobin on admission was 8.8 grams per deciliter (reference range: 13.5 - 16.5) without any known history of anemia. The remainder of the initial laboratory evaluation was unremarkable. Physical examination demonstrated right upper quadrant abdominal tenderness to palpation with a positive Murphy’s sign. Additionally, bilateral palpable supraclavicular lymph nodes were noted.

**Figure 1:**
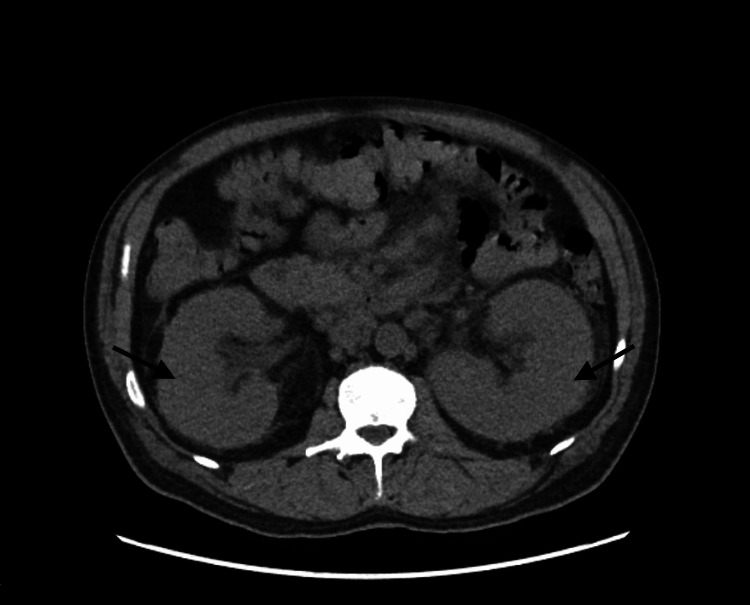
CT abdomen and pelvis without contrast (axial orientation) demonstrating severe bilateral nephromegaly measuring up to 20 cm indicated by arrows

**Figure 2:**
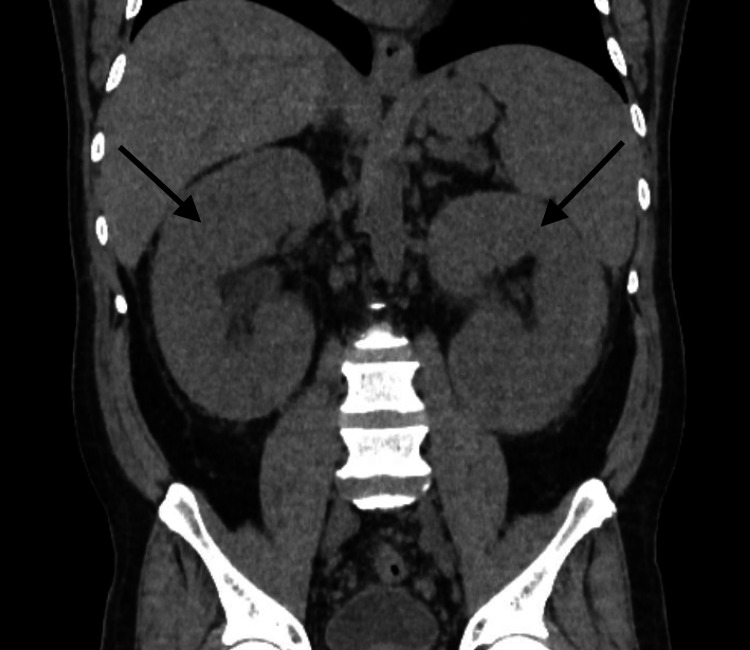
CT abdomen and pelvis without contrast (coronal orientation) demonstrating severe bilateral nephromegaly measuring up to 20 cm indicated by arrows

While in the emergency department, a point-of-care right upper quadrant abdominal ultrasound was performed and showed cholelithiasis with gallbladder wall thickening and a positive sonographic Murphy’s sign indicating cholecystitis. Subsequently, computed tomography (CT) of the abdomen and pelvis was performed and confirmed gallbladder wall thickening. There is a wide range of underlying medical conditions that can lead to gallbladder wall thickening, including cholecystitis, liver disease (hepatitis, cirrhosis, portal hypertension), extra cholecystic inflammation (pancreatitis, colitis, pyelonephritis), systemic diseases (congestive heart failure, renal failure, sepsis), and malignancy (primary gallbladder carcinoma, lymphoma) [16]. In addition to these findings, CT of the abdomen and pelvis also showed severe bilateral nephromegaly measuring up to 20 cm (Figures 1, 2), multifocal LAD (abdominal, pelvic, lower thoracic), and mild splenomegaly. Bilateral nephromegaly can be secondary to a variety of medical conditions, including diabetic nephropathy, pyelonephritis, vasculitis, renal involvement of lymphoma, polycystic kidney disease, bilateral renal cell carcinoma, renal metastases, and many more [17]. A CT scan of the chest and neck were performed and demonstrated prominent thoracic LAD and the CT neck demonstrated diffuse cervical LAD and symmetric prominence of the submandibular and parotid glands. Some of the more common underlying causes of cervical lymphadenopathy include malignancy (lymphoma, leukemia, multiple myeloma, metastatic malignancy), infection (viral, bacterial, fungal), autoimmune disease, hyperthyroidism, sarcoidosis, medication-induced, and many more [18]. Cholecystectomy was deferred pending further evaluation of LAD.

Review of a peripheral blood smear showed atypical lymphocytes with small- to medium-sized nuclei. Peripheral blood flow cytometry showed CD5+ (dim) B-cells comprising 27% of total events. B-cells were also positive for CD1d, CD20, CD38, FMC7, and monoclonal surface kappa, while they were negative for CD10, CD23, CD34, and CD200. A bone marrow biopsy was obtained, and pathology showed 13% atypical lymphocytes consistent with CD5+ B-cell lymphoma. Bone marrow flow cytometry showed 20% CD5+ (dim) lymphocytes consistent with dim CD5+ B-cell lymphoma. Additionally, fluorescence in situ hybridization (FISH) was performed on the bone marrow specimen and was positive for t(11;14). A right cervical lymph node core needle biopsy was performed, and immunohistochemical stains were positive for CD5, CD20, Cyclin D1, and BCL-2, while they were negative for CD3 and BCL-6. Ki-67 of the cervical lymph node biopsy showed a proliferation index of 20-30%. Similar to the bone marrow, FISH of the lymph node specimen was positive for t(11;14). These findings were consistent with a diagnosis of MCL. Of note, the patient’s LDH level on presentation was 198 units per liter (reference range: 100 - 210), his mantle cell lymphoma international prognostic index (MIPI) score was 6.1 indicating intermediate risk, and he was determined to have stage IV disease given his extranodal organ involvement.

After cervical lymph node and bone marrow biopsies had been completed, the patient was started on oral dexamethasone 20 mg daily for a three-day course. His kidney function improved with a decline in creatinine from 4.00 mg/dl to 2.34 mg/dl upon completion of the three-day course of steroids. He subsequently underwent laparoscopic cholecystectomy with the gallbladder specimen being sent for pathology.

**Figure 3:**
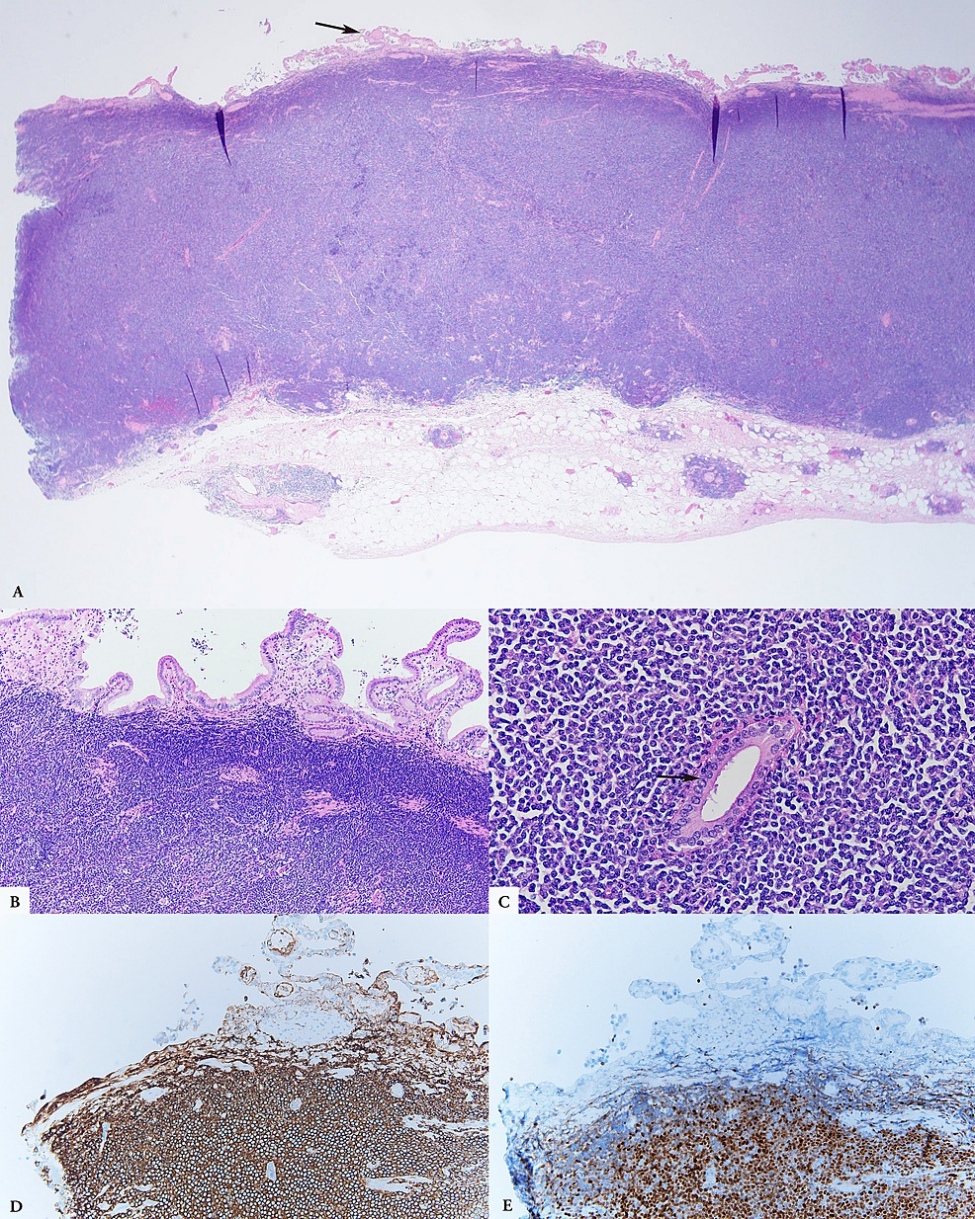
Mantle cell lymphoma involving gallbladder. H&E of mantle cell lymphoma (A, x20; B, x200; C, x400). CD20 immunohistochemical stain (D, x200). Cyclin D1 immunohistochemical stain (E, x200).

Hematoxylin and Eosin (H&E) sections show extensive lymphomatous involvement of the gallbladder with transmural infiltration and denudation of the mucosal surface (Figure 3A, indicated by arrow). In some areas of the gallbladder with remaining intact mucosal surface, lymphoma cells obliterate underlying normal anatomic structure with one intact gland as indicated by an arrow (Figure 3B, 3C). The lymphoma cells are medium in size and show irregular nuclear contour, vesicular chromatin, occasional indistinct nucleoli and scant amount of cytoplasm. Brisk mitotic figures and necrosis are not evident. CD20 and Cyclin D1 immunohistochemical stains (Figure 3D, 3E respectively) highlight lymphoma cells and confirm the diagnosis of MCL.

Following cholecystectomy, the patient completed two additional doses of oral dexamethasone 20 mg daily prior to discharge from the hospital. At the time of discharge, his creatinine had improved to 1.65 mg/dl.

After discharge, the patient established care with an outpatient hematologist for further management of his newly diagnosed MCL. Of note, his next generation sequencing testing had returned positive for MLH1 A681T variant mutation, but negative for TP53 mutation. Chemoimmunotherapy with BR (bendamustine, rituximab) was initiated, and he completed three cycles. Following this, he was transitioned to treatment with R-BAC (rituximab + bendamustine, cytarabine) to complete three additional cycles. He had been referred for hematopoietic stem cell transplantation (HSCT) evaluation in preparation for potential bone marrow transplant in the future.

## Discussion

MCL is a rare subtype of B-cell non-Hodgkin’s lymphoma, comprising between 3-10% of all non-Hodgkin’s lymphoma and having an annual incidence of one per 200,000 people [1,19]. Due to overall advancements in medical care, the average life expectancy in the general population continues to increase. Given that the median age at diagnosis of MCL ranges from 60-70 years of age, the incidence of MCL continues to rise due to the prolonged life expectancy [1,20]. MCL typically presents as advanced staged disease with widespread disease involvement at initial diagnosis [2]. The overall five-year relative survival rate of patients with MCL is around 50%, with the rate decreasing to approximately 36% in patients >75 years old [20]. Given the frequently advanced stage of MCL at the time of presentation, prompt diagnosis and treatment are crucial to improve patient outcomes [2]. While recognizing the more common presenting symptoms of lymphoma (B-symptoms, LAD, blood count abnormalities) is vital, awareness of the less common presenting symptoms is necessary to diagnose and manage patients with rare presentations in a timely manner [1].

Two of the uncommon extranodal sites of MCL involvement are the gallbladder and kidneys, as seen in our case. While our patient did not have biopsy-proven renal involvement, it was strongly suspected as there was no alternative clinical explanation, and renal function improved with dexamethasone. Given the rarity of these sites of involvement, only case reports detailing these presentations have been described in literature, leading to lack of physician awareness necessary to make a prompt diagnosis [10-15]. In this case report, the patient initially presented with cholecystitis and bilateral nephromegaly. While cholecystitis isn’t an uncommon presenting symptom for GI involvement of lymphoma, it is rare for MCL to be the underlying lymphoma subtype [7]. Additionally, renal lymphomatous involvement presenting as nephromegaly is rare in all subtypes of lymphoma, including MCL [8,9].

With a new diagnosis of MCL that is symptomatic on presentation, single-agent therapy with corticosteroids is commonly utilized to produce a temporary response [21]. However, it is paramount to obtain histologic evidence of lymphoma prior to the initiation of steroids to avoid reductions in the diagnostic yield of biopsy whenever possible. In this case report, the patient had symptomatic involvement of the gallbladder and kidneys which was initially treated with a five-day course of dexamethasone, resulting in symptomatic improvement and improvement in renal function. Once patients are clinically stabilized and a final diagnosis of MCL has been made, decisions regarding treatment with chemoimmunotherapy and/or HSCT are determined. Generally, fit patients with advanced staged, symptomatic MCL initially undergo chemoimmunotherapy [1,2]. Some commonly utilized chemoimmunotherapy regimens include R-CHOP (rituximab + cyclophosphamide, doxorubicin, vincristine, prednisone), R-DHAP (rituximab + dexamethasone, cytarabine, cisplatin), BR, and R-BAC [1,2]. Patients with advanced, fast-growing MCL commonly undergo an aggressive, intensified treatment regimen. Some commonly utilized induction treatment regimens in these scenarios include the LYMA regimen (four cycles of R-DHAP followed by R-CHOP), NORDIC regimen (R-CHOP alternating with rituximab + high-dose cytarabine), BR followed by rituximab + high-dose cytarabine, TRIANGLE regimen (R-CHOP + ibrutinib alternating with R-DHAP), and HyperCVAD + R (cyclophosphamide, vincristine, doxorubicin, dexamethasone + rituximab) [22]. The patient in this case report was initially treated with BR, followed by R-BAC, which has been shown to be an effective approach in patients with previously untreated MCL [23]. In eligible patients who achieve remission after initial induction therapy, utilization of HSCT consolidation is a commonly utilized approach in the management of MCL [1,22,24].

## Conclusions

In conclusion, we report the case of a patient who presented with cholecystitis and bilateral nephromegaly found to be secondary to newly diagnosed MCL. Given the patient had associated LAD noted on imaging and physical examination, cervical lymph node biopsy and bone marrow biopsy were completed, confirming the diagnosis of MCL. A laparoscopic cholecystectomy was performed, and gallbladder pathology was consistent with MCL involvement. Initial presentation of MCL with extranodal involvement of the gallbladder and/or kidneys is extraordinarily rare and is infrequently documented in literature. We have reported the present case study to assist in the prompt diagnosis and management of patients with atypical presentations of MCL.
